# Inference under unequal probability sampling with the Bayesian exponentially tilted empirical likelihood

**DOI:** 10.1093/biomet/asaa028

**Published:** 2020-12

**Authors:** A. Yiu, R. J. B. Goudie, B. D. M. Tom

**Affiliations:** Medical Research Council Biostatistics Unit, School of Clinical Medicine, University of Cambridge, Robinson Way, Cambridge CB2 0SR, U.K.

**Keywords:** Bayesian method of moments, Bernstein–von Mises theorem, Double robustness, Exponentially tilted empirical likelihood, M-estimation, Selection bias

## Abstract

Fully Bayesian inference in the presence of unequal probability sampling requires stronger structural assumptions on the data-generating distribution than frequentist semiparametric methods, but offers the potential for improved small-sample inference and convenient evidence synthesis. We demonstrate that the Bayesian exponentially tilted empirical likelihood can be used to combine the practical benefits of Bayesian inference with the robustness and attractive large-sample properties of frequentist approaches. Estimators defined as the solutions to unbiased estimating equations can be used to define a semiparametric model through the set of corresponding moment constraints. We prove Bernstein–von Mises theorems which show that the posterior constructed from the resulting exponentially tilted empirical likelihood becomes approximately normal, centred at the chosen estimator with matching asymptotic variance; thus, the posterior has properties analogous to those of the estimator, such as double robustness, and the frequentist coverage of any credible set will be approximately equal to its credibility. The proposed method can be used to obtain modified versions of existing estimators with improved properties, such as guarantees that the estimator lies within the parameter space. Unlike existing Bayesian proposals, our method does not prescribe a particular choice of prior or require posterior variance correction, and simulations suggest that it provides superior performance in terms of frequentist criteria.

## 1 Introduction

Consider the problem of estimating a population parameter vector in the presence of unequal probability sampling. We investigate two settings. The first, which we refer to as the design setting, assumes a selection mechanism determined by the data collector, but only partial design information in the form of sampling probabilities for the selected individuals is provided to the analyst. This situation is frequently encountered when analysing public-use survey datasets (Si et al., 2015; Zanganeh & Little, 2015; Wang et al., 2017). The second scenario is an observational setting where the selection mechanism is unknown, but assumed to be ignorable conditional on a set of fully observed covariates. Certain problems in missing data and causal inference, such as estimating an average treatment causal effect with no unmeasured confounders, can be formulated in this framework (Kang & Schafer, 2007).

In both cases, it is common in practice to use semiparametric estimators that incorporate inverse probability weighting. If the selection probabilities are known, weighting methods are simple to implement and require few modelling assumptions for consistency (Horvitz & Thompson, 1952; Hájek, 1971). In the observational setting, inverse probability weights estimated from a selection model can be combined with an imputation model to produce doubly robust estimators that are consistent as long as one of the models is correctly specified (Robins et al., 1994; Scharfstein et al., 1999; Kang & Schafer, 2007; Rotnitzky & Vansteelandt, 2014). However, while the large-sample properties of these estimators are attractive, their reliability for small datasets is less justified theoretically. In particular, the use of inverse probability weighting can lead to disastrous performance in the presence of model misspecification and a practical violation of positivity (Kang & Schafer, 2007). For small-sample inference, the prior distribution in a Bayesian approach can offer both regularization and a systematic way of incorporating informative knowledge into the analysis, with this influence gradually relaxed as the sample size increases. The Bayesian paradigm also provides a convenient framework for evidence synthesis (Ades & Sutton, 2006); repeatedly integrating over the posterior distributions of parameters allows one to propagate uncertainty across multiple data sources within a single analysis.

A significant drawback of Bayesian approaches is the requirement of stronger structural assumptions on the data distribution and the sampling mechanism. In the design setting, one option is to specify a flexible regression imputation model with the sampling probability included as a covariate to adjust for the selection bias. To obtain estimates for the target population, the sampling probability can be integrated out using a sampling probability model conditional on selection (Si et al., 2015; Zanganeh & Little, 2015). Alternatively, one could adopt a sample likelihood approach (Pfeffermann et al., 2006) which truncates the dataset to just the sampled individuals and requires the specification of a conditional selection model. Both approaches involve directly modelling the dependence structure between the incomplete data and the sampling probabilities, rather than using the unavailable design variables specified by the data collector. This is potentially difficult to specify correctly. In the observational setting, Bayesian estimators will generally fail to be doubly robust; either the selection mechanism is ignored or the model parameters are a priori dependent, so that misspecification of just one model can feed back into the other, precluding consistency (Zigler et al., 2013; Robins et al., 2015). Specializing to mean estimation of binary outcomes, Ray & van der Vaart (2019) presented a thorough treatment of assumptions and priors for fully Bayesian approaches to satisfy Bernstein–von Mises theorems, as well as the proposal of novel propensity score-dependent priors that incorporate a preliminary estimate of the propensity score into the prior of the outcome regression model.

There have been a number of Bayes-like proposals that aim to resolve these issues. In a survey inference context, Wang et al. (2017) suggested using an approximate normal likelihood centred at a weighted estimator to match the robustness and simplicity of the frequentist approach. McCandless et al. (2010) and Graham et al. (2016) achieved double robustness by cutting the feedback between the models, but this necessitates a post hoc variance correction. This idea was reviewed by Saarela et al. (2016), who also proposed a method for doubly robust estimation using a Bayesian bootstrap model. Although their method does not require variance correction and matches the performance of the frequentist estimators, it prescribes a specific choice of noninformative prior. For the estimation of average treatment effects, with a particular focus on settings where the dimension of the covariates is high relative to the sample size, Antonelli & Dominici (2019) proposed a partially Bayesian method. The resulting posterior has the double robustness property, but also requires a variance correction using a method such as the nonparametric bootstrap.

In this paper we develop an inferential framework that offers the practical benefits of Bayesian statistics described above, along with the attractive asymptotic guarantees of frequentist semiparametric estimators. Central to our method is a novel application of Bayesian exponentially tilted empirical likelihood (Schennach, 2005), an approach which forms a posterior by combining a prior with a likelihood function defined by moment conditions. We specialize to the domain of M-estimation, since many proposed semiparametric estimators (e.g., Hájek, 1971; Robins et al., 1994; Scharfstein et al., 1999; Cao et al., 2009; Rotnitzky et al., 2012) are M-estimators, and the unbiased estimating equations they solve are used to define a set of corresponding moment constraints. We prove Bernstein–von Mises theorems showing that the resulting Bayesian exponentially tilted empirical likelihood posterior becomes approximately normal, centred at the chosen estimator with matching asymptotic variance; the choice of prior is unrestricted, apart from continuity and nonzero mass in a neighbourhood of the probability limit of the estimator. Thus, the posterior shares the analogous properties of the estimator, such as double robustness and local efficiency, and the frequentist coverage of any credible set will be approximately equal to its credibility. In particular, the latter implication extends the large-sample posterior properties proved by Chib et al. (2018), and provides an interpretation of the credible sets as regularized or shrinkage estimators of confidence sets, filling a conceptual gap otherwise left empty due to the procedure not being fully Bayesian.

Additionally, we prove that a separation condition, similar to the requirement in Theorem 1 of Chib et al. (2018), is implied under standard assumptions for the consistency of M-estimators. This allows the user to avoid a potentially difficult verification. Schennach (2005) provided an interpretation of Bayesian exponentially tilted empirical likelihood which justifies its use as a Bayesian procedure. However, the conditions of this result are not satisfied in our design setting in § 2.3. We give an alternative interpretation, connecting the likelihood function with a proper likelihood arising from an exponential family of maximum-entropy distributions, and suggest that this paves the way for future work.

Our approach offers the ability to obtain modified versions of existing estimators with improved properties, even in the absence of informative priors. For example, certain proposed estimators (e.g., Cao et al., 2009) may have a nonzero probability of lying outside the parameter space, potentially leading to suboptimal finite-sample performance (Rotnitzky et al., 2012). This problem can be rectified by simply restricting the support of the prior, yielding a new estimator which is population bounded in accordance with the variation of its predecessor and has identical asymptotic behaviour. Having a posterior distribution also allows the user to have a choice of estimators, such as the mean, median or maximum a posteriori estimator, depending on the situation or the target loss function.

## 2 Proposal

### 2.1 Exponentially tilted empirical likelihood

Suppose that *D* is a random vector drawn from a distribution *P*_0_. The objective is to estimate the parameter *θ*_0_ ∈ Θ ⊂ ℝ*^m^*, which is assumed to satisfy the moment condition *E_Po_ {g(D*, *θ*_0_)} = 0, where *g* is a function mapping into ℝ^*m*^.Thus, the dimension of the moment conditionis assumed to match the dimension of the parameter. The observed data *D_i_* (*i* = 1, …, *n*) are independent and identically distributed replicates of *D* with realized values *d_i_*. An M-estimator ${\hat{\theta }}_{n}$ solves the estimating equation $n^{-1}\sum_{i=1}^{n}g\left(d_{i},\theta \right)=0$ for *θ* ∈ Θ. Many proposed estimators for unequal probability sampling problems take this form, accompanied by a set of regularity assumptions similar to the following.

#### Assumption 1

(i) The parameter space Θ of *θ* is compact, and *θ*_0_ lies in the interior of Θ and is the unique solution to *E*_*P*_0__ {*g* (*D*, *θ*)}=0.
(ii) With probability 1, there is a unique solution ${\hat{\theta }}_{n}$ to $n^{-1}\sum_{i=1}^{n}g\left(D_{i},\theta \right)=0$ for each *n*.
(iii) The variance Ω_0_ = var*p*_0_ {*g*(*D*, *θ*_0_)} is nonsingular.
(iv) The expectation $E_{P_{0}}\{\sup_{\theta \in \Theta }{\left||g(D,\theta )|\right|}_{2}^{2}\}<\infty$
(v) With probability 1, *g*(*D*, *θ*) is continuous at each *θ* ∈ Θ.
(vi) With probability 1, *g*(*D*, *θ*) is continuously differentiable with respect to *θ* in a neighbourhood ${\text{Θ}}^{\prime }$ of *θ*_0_, and $E_{P_{0}}\left\{\sup_{\theta^{\prime }\in {\text{Θ}}^{\prime }}\;||\partial_{\theta }g\left(D,\theta^{\prime }\right)|{|}_{\text{F}}\right\}<\infty$ where ∂_θ_ denotes the partial derivative with respect to *θ* and F refers to the Frobenius norm.
(vii) The matrix *G*_0_ = *E*_*P*_0__{∂θ*g*(*D*, *θ*_0_)} is invertible.

Assumption 1 is sufficient for the M-estimator ${\hat{\theta }}_{n}$ to be consistent and asymptotically normally distributed (van der Vaart, 1998),

$$n^{1/2}\left({\hat{\theta }}_{n}-\theta_{0}\right)\to N\left(0,{\text{Σ}}_{0}\right)$$

in the sense of convergence in distribution, with ${\text{Σ}}_{0}={\left(G_{0}^{\text{T}}\Omega_{0}^{-1}G_{0}\right)}^{-1}$ where *G*_0_ and Ω_0_ can be consistently estimated by

(1) $${\hat{G}}_{n}=n^{-1}\overset{n}{\underset{i=1}{\sum^{\text{​}}}}\partial_{\theta }g\left(D_{i},{\hat{\theta }}_{n}\right),\;{\hat{\Omega }}_{n}=n^{-1}\overset{n}{\underset{i=1}{\sum^{\text{​}}}}g\left(D_{i},{\hat{\theta }}_{n}\right)g{\left(D_{i},{\hat{\theta }}_{n}\right)}^{\text{T}},$$

respectively.

The moment condition *g* can also define a semiparametric model by restricting to distributions *P* that satisfy *E_P_*{*g*(*D*, *θ*)} = 0 for *θ* ∈ Θ. For values of *θ* such that the origin lies in the convex hull of {*g*(*d_i_*,*θ*): *i* = 1,…,*n*}, the exponentially tilted empirical likelihood (Jing & Wood, 1996; Corcoran, 1998; Lee & Young, 1999; Schennach, 2005) is defined, up to a constant factor, as

$$L_{n}(\theta )=\overset{n}{\underset{i=1}{\prod^{\text{​}}}}np_{i}(\theta ),$$

where the probabilities *p*_1_(θ), …, *p_n_*(θ) solve the optimization problem

(2) $$\underset{p_{1},\ldots ,p_{n}}{\max}\overset{n}{\underset{i=1}{\sum^{\text{​}}}}\left(-p_{i}\log p_{i}\right)$$

subject to

(3) $$\overset{n}{\underset{i=1}{\sum^{\text{​}}}}p_{i}=1,\;\overset{n}{\underset{i=1}{\sum^{\text{​}}}}p_{i}g\left(d_{i},\theta \right)=0,\;p_{i}⩾0\;(i=1,\ldots ,n).$$

For other values of *θ*, *L_n_*(θ) is set to 0. The function *p*(θ) ={*p*_1_(θ),…,*p_n_*(θ)}^T^ is well-defined since: (i) for each value ofθ the constraint set is compact and the objective function is continuous, so if the constraint set is nonempty then the objective function attains the maximum; and (ii) the objective function is strictly concave, so there is a unique maximizer.

One may interpret this likelihood function as being derived from a *θ*-parameterized set of multinomial distributions supported on the observed data values. For each value of *θ*, the solution minimizes the Kullback–Leibler divergence from the empirical distribution subject to the constraint *EP* {*g*(*D*, *θ*)}=0. More precisely, the Kullback–Leibler divergence is minimized with the empirical distribution as the second argument; the opposite direction corresponds to the empirical likelihood (Owen, 2001). This connection mirrors the relationship between variational Bayesian methods and expectation propagation (Gelman et al., 2013). The exponentially tilted empirical likelihood is connected to M-estimation in the following manner.

#### Proposition 1

*The M-estimator ${\hat{\theta }}_{n}$ maximizes the exponentially tilted empirical likelihood*.

Furthermore, we show that Assumption 1 is sufficient for establishing the following separation property, which says that *L_n_* decays superpolynomially to 0 outside of any ball around ${\hat{\theta }}_{n}$.

#### Theorem 1

*If Assumption 1 is satisfied, then for any* δ > 0 *there exists an* ε > 0 *such that*

$$\underset{∥\theta -{\hat{\theta }}_{n}{∥}_{2}⩾\delta }{\sup}\frac{L_{n}(\theta )}{L_{n}\left({\hat{\theta }}_{n}\right)}⩽\exp\left\{-\varepsilon {\left(n-1\right)}^{1/2}\right\}$$

*with probability approaching* 1.

### 2.2 Bayesian exponentially tilted empirical likelihood

From a Bayesian perspective, Schennach (2005) proposed that the exponentially tilted empirical likelihood could be combined with a prior *p*(θ) to form a posterior

$$p\left(\theta |d_{1},\ldots ,d_{n}\right)\propto L_{n}(\theta )p(\theta )$$

and referred to this approach as Bayesian exponentially tilted empirical likelihood. Schennach justified this by proving that if all observed data values are distinct, then *L_n_*(θ)can be represented as a limit

$$L_{n}(\theta )=\underset{\varepsilon \to 0}{\text{lim}}\;\underset{B\to \infty }{\text{lim}}\int^{\text{​}}\left\{\overset{n}{\underset{i=1}{\prod^{\text{​}}}}p\left(d_{i}|\xi_{B}\right)\right\}p\left(\xi_{B}|\theta ;\varepsilon \right)\text{d}\xi_{B},$$

which suggests that it has a proper probabilistic interpretation as a likelihood derived from a semiparametric model after marginalizing an infinite-dimensional nuisance parameter. The prior for the nuisance parameter *ξ_B_* = (*ξ*_*B*,1_, …, *ξ_B_*,_*B*_)^T^ conditional on *θ* and a positive real number ε is a distribution on a grid of values such that the induced mixture of uniform densities centred on the components of *ξ_B_* satisfies the moment restrictions with in a tolerance of ε, favouring mixtures with small support. Conditional on *ξ_B_*,*D_i_* is distributed according to the corresponding mixture of uniform densities. As *B* →∞, the spacing of the grid of values tends to zero and the range tends to infinity. Chib et al. (2018) further proved Bernstein–von Mises results, showing that the total variation distance between the posterior distribution of *n* ^1/2^(θ–θ_0_) and the normal distribution *N*(0, ∑_0_) tends to zero under correctly specified moment constraints.

We specialize to the domain of M-estimation and prove a Bernstein-von Mises theorem with centring point being the M-estimator ${\hat{\theta }}_{n}$. This implies not only that the posterior is consistent and asymptotically normal, but also that frequentist coverage of any credible set will be approximately equal to its credibility, extending the properties implied by the results of Chib et al. (2018). We specify a distinct set of further assumptions.

If *L_n_*(θ) is nonzero, the optimization problem specified by (2) and (3) can be solved (Schennach, 2007) by considering the dual problem

(4) $$p_{i}(\theta )=\frac{\exp\left\{{\hat{\lambda }}_{n}{\left(\theta \right)}^{\text{T}}g\left(d_{i},\theta \right)\right\}}{\sum_{j=1}^{n}\exp\left\{{\hat{\lambda }}_{n}{\left(\theta \right)}^{\text{T}}g\left(d_{j},\theta \right)\right\}},$$

where ${\hat{\lambda }}_{n}(\theta )$ solves

(5) $$\overset{n}{\underset{i=1}{\sum^{\text{​}}}}\exp\left\{\lambda^{\text{T}}g\left(d_{i},\theta \right)\right\}g\left(d_{i},\theta \right)=0.$$

#### Assumption 2

There exists a neighbourhood *B* of *θ*_0_ on which, with probability approaching 1, the exponentially tilted empirical likelihood is nonzero or, equivalently, there exists a function ${\hat{\lambda }}_{n}:ℬ\to {\mathbb{R}}^{m}$ such that for all *θ* ∈ *B*,

$$\overset{n}{\underset{i=1}{\sum^{\text{​}}}}\exp\left\{{\hat{\lambda }}_{n}{\left(\theta \right)}^{\text{T}}g\left(d_{i},\theta \right)\right\}g\left(d_{i},\theta \right)=0.$$

#### Assumption 3

For almost all values of *d*, *g*(*d*,θ)is twice differentiable with respect to *θ* in a neighbourhood ofθ_0_, and the second derivative satisfies the Lipschitz condition

$$∥\partial_{\theta }^{2}g(d,\theta )-\partial_{\theta }^{2}g\left(d,\theta^{\prime }\right){∥}_{\text{op }}⩽\psi (d)∥\theta -\theta^{\prime }∥2$$

for an integrable function ψ, where op refers to the operator norm.

#### Assumption 4

For almost all values of *d*, there exists a neighbourhood of (0,θ_0_) contained in ℝ*_m_* × Θ in which the function

$$f(\lambda ,\theta )=\exp\left\{\lambda^{\text{T}}g(d,\theta )\right\}g(d,\theta )$$

and all of its first and second partial derivatives are dominated by an integrable function.

These conditions allow us to establish the following intermediate result.

#### Proposition 2

*If* Assumptions 3 and 4 *are satisfied, then on a neighbourhood of θ*_0_ *there exists a unique function* *λ*_0_ *mapping into* ℝ^*m*^ *such that*

$$E_{P_{0}}\left[\exp\left\{\lambda_{0}{\left(\theta \right)}^{\text{T}}g(D,\theta )\right\}g(D,\theta )\right]=0$$

*and *λ**_0_ *is twice Lipschitz differentiable*.

Consequently, one can generate an exponential family {*P*θ } from *P*_0_ via

$$\frac{\text{d}P_{\theta }}{\text{d}P_{0}}(d)=\exp\left\{\lambda_{0}{\left(\theta \right)}^{\text{T}}g(d,\theta )-\kappa (\theta )\right\}$$

locally around *θ*_0_, where κ(θ) = log*E*_*P*_0__[exp{*λ*_0_(θ)^T^ *g*(*D*, *θ*)}] and *EP*_θ_{*g*(*D*,θ)}=0. The exponentially tilted distribution *P_θ_* is the I-projection (see Csiszár, 1975) of *P*_0_ onto the set {*P* : *EP*{*g*(*D*, *θ*)}=0}, i.e., the closest element to *P*_0_ in the set in terms of Kullback–Leibler divergence. In this local region of *θ*_0_, the exponentially tilted empirical likelihood is approximately equal to the likelihood generated by this exponential family. This suggests that the exponentially tilted empirical likelihood is a plug-in estimate of a least-favourable family of distributions aimed at reducing the original semiparametric model to a parametric model in a minimally informative way. This provides a general interpretation of the Bayesian exponentially tilted empirical likelihood approach that holds even in certain situations to which the Schennach (2005) interpretation does not apply, such as the design setting in §2.3 where the set of observed data values may not be distinct.

#### Theorem 2

*Suppose that Assumptions* 1*–*4 *hold. Assume also that the prior p*(θ) *admits a continuous density with respect to the Lebesgue measure andis positive at* *θ*_0_. *Then*

$$\int_{\Theta }\left|p\left(\theta |D_{1},\ldots ,D_{n}\right)-p_{{\hat{\theta }}_{n},n^{-1}{\text{Σ}}_{0}}(\theta )\right|\text{d}\theta \to 0$$

*in probability, where* $p{\hat{\theta }}_{n},n^{-1}{\text{Σ}}_{0}$ *is the density of* $N\left({\hat{\theta }}_{n},n^{-1}{\text{Σ}}_{0}\right)$.

By centring and scaling and using an alternative form of the total variation distance (Tsybakov, 2009), we obtain the equivalent representation

$$\underset{B}{\sup}\left|\text{pr}\left\{n^{1/2}\left(\theta -{\hat{\theta }}_{n}\right)\in B|D_{1},\ldots ,D_{n}\right\}-N\left(0,{\text{Σ}}_{0}\right)(B)\right|\to 0$$

in probability, where *B* ranges over all elements of the Borel sigma-algebra on ℝ^*m*^. Theorem 2 implies both posterior consistency and asymptotically correct frequentist coverage of credible sets. The following result confirms the first-order equivalence between the posterior mean and ${\hat{\theta }}_{n}$, establishing the validity of the methodology as a shrinkage estimation framework that can yield finite-sample gains while matching the asymptotic performance of the standard estimator.

#### Theorem 3

*Suppose* Assumptions 12013;4 *hold and that ∫‖θ*‖_2_ *p*(θ) dθ < ∞. *Let* $\theta_{n}^{*}=\int \theta \;p(\theta \;|\;d_{1},\ldots ,d_{n})$ d*θ be the Bayesian exponentially tilted empirical likelihood posterior mean*.

*Then*

$$n^{1/2}\left({\hat{\theta }}_{n}-\theta_{n}^{*}\right)\to 0,\;n^{1/2}\left(\theta_{n}^{*}-\theta_{0}\right)\to N\left(0,{\text{Σ}}_{0}\right),$$

*where the convergence is in probability and in distribution, respectively*.

### 2.3 Design setting

We first consider the estimation of a population parameter in a design setting where the selection probabilities are known for the sampled individuals. The data*D_i_* = (*R_i_Z_i_*, *R_i_*, *R_i_*π*_i_*) (*i* = 1, 2026; , *n*) are independent and identically distributed from *P*_0_; here *R_i_* is the selection indicator, which is equal to 1 if *Z_i_* is observed and 0 otherwise, π*_i_* = pr(*R_i_* = 1 | *W_i_*), and *Z_i_* and *R_i_* are conditionally independent given *W_i_*. The variables *W*_1_, …, *W_n_* are the design variables chosen by the data collector to assign sampling probabilities to individuals in the target population, but they are not included in the dataset. We make the positivity assumption that there exists a *δ* > 0 such that *π_i_* ⩾ *δ* with probability 1. The target parameter *θ*_0_ is the unique solution to *E*_*P*_0__ {*u(Z*, *θ*)} = 0 for a function *u* and *θ* ∈ Θ ⊂ ℝ^*m*^. The full-data estimating function *u* is adapted below to the estimating function *g* for the observed data, allowing us to apply Theorem 2.

*Example* 1 (Outcome mean). We have *Z* = *Y* and *u*(*Z*, *θ*) = *Y* - *θ*.

*Example* 2 (Linear regression). We have *Z* = (*Y*,*X*) and *u*(*Z*,θ) = *X* ^T^(*Y* – *X*θ).

Consider the estimator ${\hat{\theta }}_{n}$ that solves the estimating equation

$$\overset{n}{\underset{i=1}{\sum^{\text{​}}}}\frac{R_{i}}{\pi_{i}}u\left(Z_{i},\theta \right)=0.$$

To address the technicality that the sampling probabilities are provided as *R_i_*π*_i_* in the notation rather than just π*_i_*, we set *R_i_*/(*R_i_*π*_i_*) = 0 when *R_i_* = 0 so that *R_i_*/(*R_i_*π*_i_*) is equivalent to *R_i_*/π*_i_*. In the case of estimating the population outcome mean, this estimator specializes to the Hájek estimator (Hájek, 1971). For *D* = (*RY*,*R*,*R*π) ∼ *P*_0_ and *g*(*D*,θ) = *Ru*(*Z*,θ)/π,

$$\begin{array}{ll} E_{P_{0}}\{g(D,\theta )\} & =E_{W}E_{P_{0}|W}\left\{\frac{R}{\pi }u(Z,\theta )|W\right\}\\ \; & =E_{W}\left[\frac{E_{P_{0}|W}(R|W)}{\pi }E_{P_{0}|W}\{u(Z,\theta )|W\}\right]\\ \; & =E_{P_{0}}\{u(Z,\theta )\}, \end{array}$$

where we have used the conditional independence of *R* and *Z* conditional on *W* and the equality o f *E*_*P*_0__|*W* {*R* | *W*} and π. This shows that *θ*_0_ is the unique solutionto *E*_*P*_0__{*g* (*D*, *θ*)}=0. Let *L_n_*(θ) be the exponentially tilted empirical likelihood function corresponding to the moment conditions *E*_*P*_0__ {*g*(*D*, *θ*)} = 0 for *θ* ∈ Θ. The likelihood function is combined with a user-specified prior *p*(θ) to form a posterior

$$p\left(\theta |d_{1},\ldots ,d_{n}\right)\propto L_{n}(\theta )p(\theta ).$$

If Assumptions 1–4 are satisfied and *p*(θ) is continuous and nonzero around *θ*_0_, Theorem 2 implies that

$$\int_{\Theta }\left|p\left(\theta |D_{1},\ldots ,D_{n}\right)-p_{{\hat{\theta }}_{n},n^{-1}{\text{Σ}}_{0}}(\theta )\right|\text{d}\theta \to 0$$

in probability, where $p{\hat{\theta }}_{n},n^{-1}{\text{Σ}}_{0}$ is the density of $N({\hat{\theta }}_{n},n^{-1}{\text{Σ}}_{0})$ and ${\text{Σ}}_{0}=\lim_{n\to \infty }{\mathrm{var}}_{P_{0}}(n^{1/2}{\hat{\theta }}_{n})$ as described in §2.1 and §2.2. Since ${\hat{\theta }}_{n}$ is a consistent estimator of *θ*_0_, the posterior will concentrate around *θ*_0_ as *n* gets large. Furthermore, since Σ_0_ is equal to the asymptotic variance of $n^{1/2}{\hat{\theta }}_{n}$, the frequentist coverage of any credible set will be approximately equal to its credibility.

### 2.4 Observational setting

We consider estimation of a population parameter when the selection mechanism is unknown. The observed data *D_i_* = (*R_i_Z_i_*, *R_i_*, *W_i_*)(*i* = 1, …, *n*) are independent and identically distributed from *P*_0_; *Z_i_* and *R_i_* are as before, and *W_i_* is a vector of covariates observed for each *i* such that *Z_i_* and *R_i_* are conditionally independent given *W_i_*. The target parameter γ_0_ is the unique solutionto *E*_*P*_0__{*u*(*Z*,γ)} = 0 for a function *u* and values of γ belonging to a compact real subset Γ. In a missing data context, the conditional independence of *Z_i_* and *R_i_* is sometimes referred to as a missing-at-random assumption. This set-up may also be viewed as one arm of a point exposure causal inference problem in the potential outcomes framework, with the conditional independence corresponding to an assumption of no unmeasured confounders.

Let π_0_(*W*) = pr(*R* = 1 | *W*) be the true propensity score and let φ_0_(*W*, γ) = *E*_*P*_0__{*u*(*Z*, γ) | *W*}. We make the positivity assumption that there exists a δ > 0 such that *π_0_(W*) ⩾ δ with probability 1. Solving

$$\frac{1}{n}\overset{n}{\underset{i=1}{\sum^{\text{​}}}}\left[\frac{R_{i}u\left(Z_{i},\gamma \right)}{\hat{\pi }\left(W_{i}\right)}-\hat{\phi }\left(W_{i},\gamma \right)\left\{\frac{R_{i}}{\hat{\pi }\left(W_{i}\right)}-1\right\}\right]=0,$$

where $\hat{\pi }$ and $\hat{\phi }$ are estimators of π_0_ and φ_0_, respectively, leads to a doubly robust estimator of Y; that is, the estimator is consistent and asymptotically normal as long as at least one of $\hat{\pi }$ and $\hat{\phi }$ is consistent. There is a significant body of work regarding choices for $\hat{\pi }$ and $\hat{\phi }$, particularly for population outcome mean estimation, which lead to various favourable efficiency properties. See Kang & Schafer (2007) and Rotnitzky & Vansteelandt (2014) for comprehensive reviews.

If $\hat{\pi }$ and $\hat{\phi }$ are derived from the solutions to unbiased estimating equations, as is often the case in practice, one can exploit this to formulate a set of nested moment constraints for an exponentially tilted empirical likelihood model. We show in Theorem 4 that the resulting marginal posterior distribution of γ is calibrated asymptotically to the behaviour of the selected estimator.

We restrict our attention to parametric working models π (*W*; *α*) and φ (*W*, γ ; *β*) for real-valued parameters *α* and *β*. Suppose that $\left({\hat{\alpha }}_{n},{\hat{\beta }}_{n},{\hat{\rho }}_{n}\right)$ solve the unbiased estimating equation

$$\frac{1}{n}\overset{n}{\underset{i=1}{\sum^{\text{​}}}}U_{\alpha ,\beta ,\rho }\left(D_{i},\alpha ,\beta ,\rho \right)=0,$$

where ρ is a set of additional auxiliary parameters, possibly empty (Rotnitzky & Vansteelandt, 2014). The two parameters (*α*, *β*) can be estimated either separately or together. For example, in the case of mean estimation, Robins et al. (1994) estimated *α* with maximum likelihood for a logistic regression model, and estimated *β* separately using ordinary least squares. Scharfstein et al. (1999) also used maximum likelihood estimation for *α*, but included the reciprocal of the propensity score as a covariate in the outcome regression model.

Let ${\hat{\gamma }}_{n}$ be the solution to

$$\frac{1}{n}\overset{n}{\underset{i=1}{\sum^{\text{​}}}}\left[\frac{R_{i}u\left(Z_{i},\gamma \right)}{\pi \left(W_{i},{\hat{\alpha }}_{n}\right)}-\phi \left(W_{i},\gamma ;{\hat{\beta }}_{n}\right)\left\{\frac{R_{i}}{\pi \left(W_{i},{\hat{\alpha }}_{n}\right)}-1\right\}\right]=0.$$

Let *θ* = (*α*,*β*,ρ,γ) and define *g*(*D*,θ)={*U*_*α*,*β*,ρ_(*D*,*α*,*β*,ρ)^T^, *h*(*D*,*α*,*β*,γ)^T^}^T^, where

$$h(D,\alpha ,\beta ,\gamma )=\frac{Ru(Z,\gamma )}{\pi (W;\alpha )}-\phi (W,\gamma ;\beta )\left\{\frac{R}{\pi (W;\alpha )}-1\right\}.$$

In accordance with Assumption 1(i), we assume that there exists a value *θ*_0_ = (*α*_0_, *β*_0_, ρ_0_, γ*) which is the unique solution to *E*_*P*_0__{*g*(*D*, *θ*)}=0. We say that the working model for the propensity score is correctly specified if π_0_(*W*) = π(*W*; *α*_0_), and similarly we say that the model for φ is correctly specified if φ_0_(*W*,γ)= φ(*W*,γ;*β*_0_). If at least one of the models is correctly specified, then γ* = γ_0_ and the M-estimator ${\hat{\gamma }}_{n}$ consistently estimates the true parameter.

Let*L_n_* (θ) be the exponentially tilted empirical likelihood function corresponding to the moment conditions *EP*{*g*(*D*,θ)} = 0. The likelihood function is combined with a user-specified prior *p*(θ) to form a posterior

$$p\left(\theta |d_{1},\ldots ,d_{n}\right)\propto L_{n}(\theta )p(\theta ).$$

Let *p*(γ | *d*1, …, *dn*) be the marginal posterior for γ.

#### Theorem 4

*Suppose* Assumptions 1–4 *hold and that the prior p*(θ) *admits a continuous density with respect to Lebesgue measure and is positive at* *θ*_0_ *. Then, as n* →∞,

$$\int_{\Gamma }\left|p\left(\gamma |D_{1},\ldots ,D_{n}\right)-p_{{\hat{\gamma }}_{n},n^{-1}V_{0}}(\gamma )\right|\text{d}\gamma \to 0$$

in P_0_-probability, where $p_{{\hat{\gamma }}_{n},n^{-1}\;V_{0}}$ is the density of $N({\hat{\gamma }}_{n},n^{-1}V_{0})$ *and* $V_{0}=\lim_{n\to \infty }{\mathrm{var}}_{P_{0}}\left(n^{1/2}{\hat{\gamma }}_{n}\right)$.

As stated earlier, ${\hat{\gamma }}_{n}$ is, by construction, consistent for estimating γ_0_ provided either π_0_ (*W*) = π(*W*; *α*_0_) or φ_0_(*W*, γ) = φ(*W*, γ ; *β*_0_) for all γ or both. Therefore, Theorem 4 implies that the exponentially tilted empirical likelihood posterior shares this double robustness property; the posterior will concentrate around the true value as long as one of the working models is correctly specified. Furthermore, credible sets for γ will asymptotically have nominal frequentist coverage if consistency holds, even if one of the working models is misspecified. If both models are misspecified, the credible sets will have approximately nominal coverage for the probability limit of ${\hat{\gamma }}_{n}$, which is possibly different from γ_0_.

### 2.5 Implementation

In this subsection we describe how one can compute *L_n_*(θ) for a fixed value of *θ*. To simplify the notation, write *g_i_* = *g*(*D_i_*, *θ*) for each *i* = 1, …, *n*, suppressing the dependence on *θ*. To check whether the feasible set of the optimization problem specified by (2) and (3) is nonempty, it is sufficient and computationally convenient, for example by using an R (R Development Core Team, 2020) package such as lpSolve (Berkelaar, 2015), to check whether there exists a feasible solution to the linear programming problem

(6) $$\begin{array}{ll} \text{maximize} & 0\text{ over }\left\{x\in {\mathbb{R}}^{n}:0⩽x_{i}⩽1,i=1,\ldots ,n\right\}\\ \text{subject to} & g^{\text{T}}x=0\text{ and }c^{\text{T}}x=1, \end{array}$$

where *g* = (*g*_1_,…, *g_n_*) and *c* = (1,…,1)^T^. The objective 0 is suggested here for computational simplicity, but can be replaced by *b* ^T^ *x* for arbitrary *b* ∈ ℝ^*n*^ as we are concerned only with the feasible set. If the feasible set is empty, *L_n_*(θ) is set to zero. Otherwise, assuming that the solution to (2) and (3) lies in the interior of the simplex, i.e., all the values of *p_i_* are nonzero, the optimization problem can be solved by considering the dual problem described by (4) and (5).

Assuming that $\sum_{i=1}^{n}g_{i}g_{i}^{\text{T}}$ is strictly positive definite, a unique solution to (5) exists and can be found via the Newton–Raphson method. This requires specifying a small convergence tolerance value with respect to a norm of choice. Pseudo-code for evaluating *L_n_*(θ) is provided in the Supplementary Material. Once we are able to evaluate *L_n_* pointwise, we can perform posterior inference using standard Bayesian computational machinery such as Markov chain Monte Carlo or importance sampling.

## 3 Simulations

### 3.1 Mean estimation for binary outcomes

In this simulation, we examine estimation of the population mean of binary outcomes in a design setting. In the notation of § 2.3, *Z* = *Y*, *u*(*Z*, *θ*) = *Y* - *θ* and *θ*_0_ = *E*_*P*_0__(*Y*). The design variables *W_i_* (*i* = 1, …, *n*) are independent and identically distributed according to the beta distribution Be(1.5, 3.5), and the outcomes *Y_i_* | *W_i_* ∼ Ber(*W_i_*) so that *θ*_0_ = 0.3. The selection variables *R_i_* (*i* = 1, …, *n*) are independent and identically distributed according to *R_i_* | π*_i_* ∼ Ber(π*_i_*), where logit(π*_i_*) = *W_i_*. Thus, *Y_i_* and the selection probability π*_i_* are positively correlated, and the selection must be adjusted for in order to estimate *θ*_0_. The data available for analysis are *D_i_* = (*R_i_Y_i_*, *R_i_*, *R_i_*π*_i_*)(*_i_* = 1, …, *n*), so that the design variables are excluded.

Following the approach in §2.3, the M-estimator ${\hat{\theta }}_{n}$ is the Hájek estimator which solves

$$\overset{n}{\underset{i=1}{\sum^{\text{​}}}}g\left(D_{i},\theta \right)=\overset{n}{\underset{i=1}{\sum^{\text{​}}}}\frac{R_{i}}{\pi_{i}}\left(Y_{i}-\theta \right)=0.$$

We use *g* to define the exponentially tilted empirical likelihood *L_n_*(θ), which we combine with three different priors for *θ*: *θ* ∼ Be(0.5, 0.5), *θ* ∼ Un(0, 1) and *θ* ∼ Be(1.5, 3.5). The first is Jeffrey’s prior. The mean of the Be(1.5, 3.5) prior is equal to *θ*_0_, so we consider this prior informative, while the first two are considered noninformative.

We compare this approach with the proposal inWang et al. (2017, §2). In a survey inference context, Wang et al. (2017) suggested using a Bayesian approach with an approximate normal likelihood

$$\hat{\theta }|\theta ∼N(\theta ,\hat{V}).$$

where $\hat{\theta }$ is a consistent and asymptotically normal estimator of *θ*_0_ and $\hat{V}$ is a robust estimator of the variance of $\hat{\theta }$. The estimator $\hat{\theta }$ acts as asummary statistic for the data, such that the posterior is

$$p(\theta |\hat{\theta })\propto p(\hat{\theta }|\theta )p(\theta ),$$

where $p(\hat{\theta }|\theta )$ is defined by the normal model above and *p*(θ) is a prior for *θ*. We choose $\hat{\theta }$ to be the Hájek estimator defined above and estimate its variance with the nonparametric bootstrap. We use the same priors as defined above.

Table 1 compares the frequentist estimator ${\hat{\theta }}_{n}$ with the Bayesian methods. Each setting was replicated 2000 times. The Bayes point estimators are the posterior means. Coverage rates were computed based on central 95% credible regions. The Bayesian computation was carried out using importance sampling with 5000 particles for each replication, and the tolerance for computing the exponentially tilted empirical likelihood was 10^−4^. On a standard quad-core laptop computer, a single replication of the Bayesian exponentially tilted empirical likelihood method with *n* = 100 took 0.25 seconds to run, while the method ofWang et al. (2017) took 0.04 seconds.

The Bayesian exponentially tilted empirical likelihood estimators generally have a higher magnitude of bias than the normal approximation when a noninformative prior is used, but have lower bias with the informative prior. This reflects the fact that the exponentially tilted empirical likelihood is less informative than the normal likelihood, resulting in higher shrinkage towards the prior mean. In the case of the two noninformative priors, this causes an upward bias towards the prior mean 0.5. This conservative characteristic leads to the Bayesian exponentially tilted empirical likelihood approach having superior performance in terms of root mean squared error and coverage rate across almost all settings, particularly with the smaller sample sizes for which the normal approximation is less accurate.

### 3.2 Doubly robust mean estimation with missing data

This simulation is conducted under the observational setting described in § 2.4 and follows the design of Kang & Schafer (2007). For each *_i_* = 1, …, *n*, the vector of covariates *W_i_* = (*W_i_*_1_,*W_i_*_2_,*W_i_*_3_,*W_i_*_4_) ∼ *N*(0,*I*_4_) where *I*_4_ is the 4 ×4 identity matrix, the selection indicator *R_i_* | *W_i_* ∼ Ber{π0(*W_i_*)} where

$$\pi_{0}\left(W_{i}\right)=expit\left(\alpha_{0,1}+\alpha_{0,2}^{\text{T}}W_{i}\right),\;\alpha_{0,1}=0,\;\alpha_{0,2}=(-1,0.5,-0.25,-0.1{)}^{\text{T}},$$

and *Z* = *Y*, the outcome, with *Y_i_* | *W_i_* ∼ *N*{*m*_0_(*W_i_*),1} where

$$m_{0}\left(W_{i}\right)=\beta_{0,1}+\beta_{0,2}^{\text{T}}W_{i},\;\beta_{0,1}=210,\;\beta_{0,2}=(27.4,13.7,13.7,13.7{)}^{\text{T}}.$$

Clearly, *Y_i_* and *R_i_* are conditionally independent given *W_i_*. The data are *D_i_* = (*R_i_Y_i_*, *R_i_*, *W_i_*) (*i* = 1,…,*n*). In addition to the correctly specified models (a) $\pi (w;\alpha )=pr(R=1|W=w;\alpha )=\text{expit}(\alpha_{1}+\alpha_{2}^{\text{T}}w)$ and (b) $m(w;\beta )=E_{P_{0}}(Y|W=w;\beta )=\beta_{1}+\beta_{2}^{\text{T}}w$, we also consider the misspecified models (c) $\pi \left(w^{\prime };\alpha \right)=\text{pr}\left(R=1|W^{\prime }=w^{\prime };\alpha \right)=\text{expit}\left(\alpha_{1}+\alpha_{2}^{\text{T}}w^{\prime }\right)$ and (d) $m\left(w^{\prime };\beta \right)=E_{P_{0}}\left(Y|W^{\prime }=w^{\prime };\beta \right)=\beta_{1}+\beta_{2}^{\text{T}}w^{\prime }$, where $W_{i}^{\prime }=\left(W_{i1}^{\prime },W_{i2}^{\prime },W_{i3}^{\prime },W_{i4}^{\prime }\right)$ are transformed covariates with $W_{i1}^{\prime }=\exp\left(W_{i1}/2\right),W_{i2}^{\prime }=W_{i2}/\left\{1+\exp\left(W_{i1}\right)\right\}+10,W_{i3}^{\prime }={\left\{\left(W_{i1}W_{i3}\right)/25+0.6\right\}}^{3}$ and $W_{i4}^{\prime }={\left(W_{i2}+W_{i4}+20\right)}^{3}$. The target parameter is *μ*_0_ = *E*_*P*_0__(*Y*) = 210. We write *m* and *μ* instead of the *φ* and *γ* used in §2.4 to match the notation of Kang & Schafer (2007). For the sake of brevity, the estimators and methods described in the rest of this subsection will be expressed in terms of the correct covariates *W_i_*. Under misspecification, the covariates *W__i__* are replaced with $W_{i}^{\prime }$ as appropriate.

The doubly robust augmented inverse probability weighted estimator (Robins et al., 1994), sometimes referred to as the standard doubly robust estimator, is

(7) $${\hat{\mu }}_{\text{DR}}=\overset{n}{\underset{i=1}{\sum^{\text{​}}}}\frac{1}{n}\left[\frac{R_{i}Y_{i}}{\pi \left(W_{i};{\hat{\alpha }}_{n}\right)}-m\left(W_{i};{\hat{\beta }}_{n}\right)\left\{\frac{R_{i}}{\pi \left(W_{i};{\hat{\alpha }}_{n}\right)}-1\right\}\right]$$

with ${\hat{\alpha }}_{n}$ and ${\hat{\beta }}_{n}$ estimated via maximum likelihood estimation or, equivalently, by solving

(8) $$\frac{1}{n}\overset{n}{\underset{i=1}{\sum^{\text{​}}}}U_{\alpha }\left(D_{i},\alpha \right)=0,\;\frac{1}{n}\overset{n}{\underset{i=1}{\sum^{\text{​}}}}U_{\beta }\left(D_{i},\beta \right)=0,$$

where *U*_*α*_ and *U*_*β*_ are the score equations for the logistic and linear regression models, respectively. In this case, the set of additional auxiliary parameters ρ referred to in §2.4 is empty.

Saarela et al. (2016) proposed a Bayesian doubly robust approach using the Bayesian bootstrap (Rubin, 1981). A Dirichlet process model is specified for *D_i_* in the limit of the concentration measure tending to 0. Inference for *μ* is based on a posterior predictive distribution induced by maximizing expected utility functions. Here, we follow the approach detailed in §6.2 of their paper and choose the utility functions to match the specification of ${\hat{\mu }}_{\text{DR}}$. More explicitly, the parameters *α* and *β* are linked to the Bayesian bootstrap model via

$$\alpha =\underset{\alpha }{\arg\max}E\left\{R\left(\alpha_{1}+\alpha_{2}^{\text{T}}W\right)+\log expit\left(\alpha_{1}+\alpha_{2}^{\text{T}}W\right)\right\},$$

$$\beta =\underset{\beta }{\arg\max}E\left\{R{\left(Y-\beta_{1}-\beta_{2}^{\text{T}}W\right)}^{2}\right\},$$

corresponding to maximization of the expected loglikelihoods of, respectively, the propensity score and the outcome regression models under the posterior. The target parameter *μ* is defined by

$$\mu =E\left[\frac{RY}{\pi (W,\alpha )}-m(W,\beta )\left\{\frac{R}{\pi (W,\alpha )}-1\right\}\right].$$

In practice, we sample from the posterior predictive distribution by repeatedly generating uniform Dirichlet weights *ω* = (*ω*_1_, …, *ω_n_*) and computing ${\hat{\mu }}_{\text{DR}}$ with the fixed uniform weights (1/*n*,…,1/*n*) replaced by ω in (7) and (8). Define ${\hat{\mu }}_{\text{Sa}}$ to be the posterior predictive mean of*μ* for this method. The Bayesian exponentially tilted empirical likelihood posterior for *θ* = (**α**, **β**, **μ**) is obtained by setting *u*(*Z*, *μ*) = *Y* – *μ* and following the approach described in §2.4. We compare this with the doubly robust augmented inverse probability weighted estimator and the proposal of Saarela et al. (2016).

In Table 2, for both of the Bayesian exponentially tilted empirical likelihood estimators, we use independent, weakly informative generalized Student-*t* priors for the working models (a)–(d), all with three degrees of freedom: (a)*α* ∼ *t*_3_{(0,0,0,0,0)^T^,*I*_5_} (b)**β** ∼ *t*_3_{(210,30,10,10,10)^T^,*I*_5_}, (c) *α* ∼ *t*_3_{(0, 0, 0, 0, 0)^T^, *I*_5_}, and (d) *β* ∼ *t*_3_{(35, 50, 0, −135, 0)^T^, *I*_5_}, where the shape matrices are the 5 × 5 identity matrix so that the prior variance of each component is equal to 3. For ${\hat{\mu }}_{\text{BETEL },1}$, a weakly informative prior *μ* ∼ *t*_3_(210, 1) is specified for the target parameter, while ${\hat{\mu }}_{\text{BETEL },2}$ is equipped with a more informative prior, *μ* ~ *N*(210,1). The three parameters (*α*,*β*,*μ*) are a priori independent across all settings. Sampling from the Saarela et al. (2016) posterior can be implemented directly, as described above. The exponentially tilted empirical likelihood was computed with a tolerance of 10^-4^, and posterior samples were drawn using a Metropolis–Hastings algorithm with 2000 iterations, with an initial burn-in of 500 iterations. On a standard quad-core laptop computer, a single replication using the Bayesian exponentially tilted empirical likelihood method without parallelization took 55 seconds. Computing the frequentist estimator took 0.05 seconds, and the method of Saarela et al. (2016) took 3.71 seconds.

The results in Table 2 show that in all settings, ${\hat{\mu }}_{\text{BETEL },2}$ outperforms both ${\hat{\mu }}_{\text{DR}}$ and ${\hat{\mu }}_{\text{Sa}}$ in terms of root mean squared error and median absolute error. This illustrates that when substantial prior knowledge of the target parameter is available, use of this information in our proposed approach leads to better overall performance than the other estimators evaluated. When at least one of the working models is correctly specified, ${\hat{\mu }}_{\text{BETEL },1}$ exhibits a higher magnitude of bias than ${\hat{\mu }}_{\text{DR}}$, but lower root mean squared error and median absolute error, demonstrating the regularization benefits of using weakly informative priors with our proposal. When both working models are misspecified, the improvements are more substantial, suggesting that the use of a prior can help to alleviate the effects of misspecification. The patterns of results for ${\hat{\mu }}_{\text{BETEL },1}$ compared with ${\hat{\mu }}_{\text{Sa}}$ are less clear for the bias and root mean squared error metrics, but ${\hat{\mu }}_{\text{BETEL },1}$ has consistently lower median absolute error.

## 4 Application

We examine the association between blood pressure and sodium, and potassium consumption using data from the National Health and Nutrition Examination Survey 2003–2006. The dataset includes 13 957 individuals with full data on the relevant information who were drawn from the U.S. civilian population, which we have assumed to be constant at 300 million during the time period 2003–2006. Each observation is associated with a weight variable that is assumed to be proportional to the reciprocal of the sampling probability of the individual. This follows the example found in Lumley (2010, § 5.2.4).

We work in the design setting described in § 2.3. The aim is to fit a linear regression model for blood pressure *Y* on sodium consumption *X*_1_ and potassium consumption *X*_2_. Age *X*_3_ is also included for deconfounding. The moment condition is

$$g(D,\theta )=RW\left(Y-\theta_{0}-X_{1}\theta_{1}-X_{2}\theta_{2}-X_{3}\theta_{3}\right)X,$$

where *R* is the selection indicator variable, *W* is the weight variable and *X* = (1,*X*_1_,*X*_2_,*X*_3_)^T^. We consider the frequentist M-estimator with standard errors estimated using the sandwich estimator (1). For our Bayesian exponentially tilted empirical likelihood proposal, each regression parameter is assigned an independent prior: *θ*_0_ ∼ *t*_3_(100,1), *θ*_1_ and *θ*_2_ follow half-normal distributions on the positive and negative real numbers, respectively, each with scale parameter 1, and *θ*_3_ ∼ *t*_3_(0, 1). The priors for *θ*_1_ and *θ*_2_ reflect the substantial prior evidence that sodium raises blood pressure in humans while potassium does the opposite. The likelihood was computed with a tolerance of 10^-4^, and posterior samples were drawn using a Metropolis–Hastings algorithm.

Table 3 compares the frequentist estimates with the Bayesian exponentially tilted empirical likelihood posterior mean estimates. Besides the analysis of the full dataset, an analysis of a random sample of 300 samples was also carried out. With the smaller dataset, the frequentist approach leads to a positive estimated value for the effect of potassium on blood pressure. On the other hand, the Bayesian exponentially tilted empirical likelihood approach gives an estimated value much closer to the values obtained from the full dataset, illustrating the potentially significant impact of using an informative prior. The results of both approaches converge as the sample size increases, in accordance with our theory.

## 5 Discussion

Empirical likelihood estimators for missing data problems have previously been proposed by Qin & Zhang (2007) and Chan & Yam (2014). Their work provides a convenient framework for integrating multiple working models into a single analysis, extending the doubly robust property to a multiply robust one. The methods are based on maximizing the conditional empirical likelihood of the outcomes and covariates given selection, and thus differ from ours, which uses the marginal exponentially tilted empirical likelihood.

Lazar (2003) provides a justification for using the empirical likelihood for Bayesian inference based on a criterion proposed by Monahan&Boos (1992). We expect a corresponding justification to hold for the exponentially tilted empirical likelihood because of their similarity. Previous theoretical and empirical comparisons suggest that, in addition to the empirical likelihood, other approximate likelihoods such as the bootstrap likelihood (Davison et al., 1992) and the implied likelihood (Efron, 1993) would yield similar performance.

As suggested in §2.1, the empirical distribution may be viewed as an initial estimate of the true data-generating distribution in the exponentially tilted empirical likelihood. From this perspective, it is natural to wonder whether this initial estimate can be improved. While the empirical distribution can be applied very generally, its use may disregard additional known or assumed structure about the data distribution, such as its support, conditional independencies and smoothness. Nonparametric techniques such as density estimation may offer a way to incorporate this information into the initial estimate. Investigating whether such replacements are advantageous is a topic of further research.

## Supplementary Material

Supplementary material available at *Biometrika* online includes pseudo-code for the implementation and proofs of all the theoretical results.

Supplementary File

## Figures and Tables

**Table 1:** Bias, root mean squared error and coverage rate from 2000 Monte Carlo simulations using the Hájek estimator, the normal approximation of Wang et al. (2017) and the proposed Bayesian exponentially tilted empirical likelihood approach

Population size	Prior	Method	Bias (×100)	RMSE (×100)	CR (%)
*n* = 25		Hájek	0.17	11.67	
	Jeffrey’s	Normal BETEL	-1.57 1.19	13.06 11.79	88.6 92.9
	Uniform	Normal BETEL	0.72 2.30	11.55 11.06	91.5 94.5
	Be(1.5, 3.5)	Normal BETEL	-1.62 -0.11	10.22 9.18	92.3 96.2
*n* = 50		Hájek	0.08	8.27	
	Jeffrey’s	Normal BETEL	-0.69 0.88	8.92 8.29	92.1 94.7
	Uniform	Normal BETEL	0.39 1.45	8.28 8.18	91.7 94.6
	Be(1.5, 3.5)	Normal BETEL	-1.06 0.11	7.32 7.33	92.6 95.7
*n*= 100		Hájek	-0.08	6.01	
	Jeffrey’s	Normal BETEL	-0.23 0.51	6.24 6.03	92.1 94.8
	Uniform	Normal BETEL	-0.11 0.57	6.03 5.87	92.5 94.6
	Be(1.5, 3.5)	Normal BETEL	-0.75 -0.08	5.75 5.56	92.8 94.9

RMSE, root mean squared error; CR, coverage rate of 95% credible regions; BETEL, Bayesian exponentially tilted empirical likelihood.

**Table 2:** Monte Carlo simulations based on 1000 replications using the standarddoublyrobust estimator, the methodofSaarela et al. (2016) andthe Bayesian exponentially tilted empirical likelihood approach

OR correct, PS correct					OR incorrect, PS correct				
Estimator	Bias	RMSE	MAE	ESD	Estimator	Bias	RMSE	MAE	ESD
${\hat{\mu }}_{\text{DR}}$	−0.01	2.55	1.73	2.55	${\hat{\mu }}_{\text{DR}}$	0.27	3.61	2.32	3.60
${\hat{\mu }}_{\text{Sa}}$	0.01	2.57	1.71	2.57	${\hat{\mu }}_{\text{Sa}}$	0.57	3.44	2.31	3.39
${\hat{\mu }}_{\text{BETEL },1}$	0.04	2.23	1.24	2.23	${\hat{\mu }}_{\text{BETEL },1}$	0.31	3.55	2.13	3.54
${\hat{\mu }}_{\text{BETEL },2}$	−0.05	1.95	1.18	1.95	${\hat{\mu }}_{\text{BETEL },2}$	0.29	2.87	1.81	2.85
OR correct, PS incorrect					OR incorrect, PS incorrect				
Estimator	Bias	RMSE	MAE	ESD	Estimator	Bias	RMSE	MAE	ESD
${\hat{\mu }}_{\text{DR}}$	−0.01	2.59	1.73	2.59	${\hat{\mu }}_{\text{DR}}$	−6.44	38.52	3.64	37.97
${\hat{\mu }}_{\text{Sa}}$	−0.09	2.60	1.73	2.60	${\hat{\mu }}_{\text{Sa}}$	−4.81	15.41	3.38	14.64
${\hat{\mu }}_{\text{BETEL },1}$	0.06	2.32	1.33	2.32	${\hat{\mu }}_{\text{BETEL },1}$	−5.11	14.75	3.36	13.84
${\hat{\mu }}_{\text{BETEL },2}$	−0.09	2.10	1.29	2.10	${\hat{\mu }}_{\text{BETEL },2}$	−2.37	4.20	2.51	3.47

RMSE, root mean squared error; MAE, median of absolute errors; ESD, empirical standard deviation; DR, doubly robust; Sa, the method of Saarela et al. (2016); BETEL, Bayesian exponentially tilted empirical likelihood; OR, outcome regression; PS, propensity score; OR correct, use of the correct outcome regression model (a); OR incorrect, use of the model (c); PS correct, use of the correct propensity score model (b); PS incorrect, use of the model (d).

**Table 3:** Frequentist estimates and standard errors compared with Bayesian exponentially tilted empirical likelihood posterior means and posterior standard deviations

Sample size	Method		*θ*_0_	*θ*_1_	*θ*_2_	*θ*_3_
*n* = 300	Frequentist	Estimate	95.10	0.39	0.85	0.54
		Standard error	2.85	0.63	0.94	0.04
	BETEL	Posterior mean	99.31	0.51	-0.56	0.52
		Posterior s.d.	1.22	0.29	0.39	0.03
*n* = 13 957	Frequentist	Estimate	99.74	0.80	-0.91	0.50
		Standard error	0.80	0.15	0.19	0.01
	BETEL	Posterior mean	99.82	0.78	-0.89	0.49
		Posterior s.d.	0.39	0.09	0.12	0.01

BETEL, Bayesian exponentially tilted empirical likelihood; s.d., standard deviation.
