# Supplementary material for: Inference under unequal probability sampling with the Bayesian exponentially tilted empirical likelihood
Source: Biometrika. Author manuscript; Available in PMC 2022 Jan 5. (PMC7612173; doi:10.1093/biomet/asaa028)
Supplement: Supplementary File [file EMS140643-supplement-Supplementary_File.pdf]

Biometrika (2019), *pp.* 1–10
Printed in Great Britain
Advance Access publication

Supplementary material for ‘Inference under unequal probability sampling with the Bayesian exponentially tilted empirical likelihood’

BY A. YIU, R. J. B. GOUDIE AND B. D. M. TOM
Medical Research Council Biostatistics Unit, School of Clinical Medicine, University of Cambridge, Robinson Way, Cambridge CB2 0SR, U.K.
andrew.yiu@mrc-bsu.cam.ac.uk robert.goudie@mrc-bsu.cam.ac.uk
brian.tom@mrc-bsu.cam.ac.uk

SUMMARY

This supplementary material contains pseudo-code for the implementation of our approach, as well as proofs of all stated results.

1. IMPLEMENTATION PSEUDO-CODE

Further to our description in §2.5, we provide pseudo-code of the likelihood computation algorithm to assist users in implementing the method.

Define the Hessian matrix function

$$H(\lambda) = \sum_{i=1}^{n} \exp\{\lambda^{\mathrm{T}} g_i\} g_i g_i^{\mathrm{T}}.$$

Algorithm 1. Computing the exponentially tilted empirical likelihood

input θ and τ_0
Solve linear programming problem described by (9) in the main text
If no feasible solutions exist
    output 0
else
    $\lambda \leftarrow (0, \ldots, 0)$
    $\tau \leftarrow \tau_0 + 1$
    while $\tau > \tau_0$
        $s \leftarrow H(\lambda)^{-1} f(\lambda)$
        $r \leftarrow 0$
        $\lambda' \leftarrow \lambda - s$
        while $f(\lambda) > f(\lambda')$
            $r \leftarrow r + 1$
            $\lambda' \leftarrow \lambda - 2^{-r} s$
        $\tau \leftarrow \|\lambda' - \lambda\|$
        $\lambda \leftarrow \lambda'$
    for $i = 1$ to $i = n$
        $p_i \leftarrow \exp(\lambda^{\mathrm{T}} g_i) / \sum_{j=1}^{n} \exp(\lambda^{\mathrm{T}} g_j)$
    $L \leftarrow \prod_{i=1}^{n} n p_i$
    output L

© 2019 Biometrika Trust

2. NOTATION

To reduce the amount of notational clutter in the proofs, we introduce the notation (i) $l_n(\theta) = \log L_n(\theta)$ and (ii) $g_i(\theta) = g(d_i, \theta)$.

3. PROOFS

Proof of Proposition 1. The optimization problem

$$\max_{p_1,\dots,p_n} \sum_{i=1}^{n} \{-p_i \log p_i\}$$

subject to

$$\sum_{i=1}^{n} p_i = 1$$

is solved uniquely by $p_i = 1/n$ for each $i = 1, \dots, n$ (using the method of Lagrange multipliers for example). If the additional constraint

$$\sum_{i=1}^{n} p_i g(d_i, \hat{\theta}_n) = 0$$

is imposed, it follows that $p_i = 1/n$ for each $i = 1, \dots, n$ is still the unique solution since it satisfies the constraint. By the AM-GM inequality,

$$L_n(\theta) = \prod_{i=1}^{n} n p_i(\theta) \leq 1$$

with equality if and only if each $p_i(\theta)$ is equal to $1/n$, attained at $\theta = \hat{\theta}_n$. □

Proof of Theorem 1. From the proof of Proposition 1, $L_n(\hat{\theta}_n) = 1$. Furthermore, by consistency of $\hat{\theta}_n$, θ_0 will lie in the ball $\{\theta : \|\theta - \hat{\theta}_n\| \leq \delta/2\}$ with probability approaching one. Hence,

$$\sup_{\|\theta-\hat{\theta}_n\|_2 \geq \delta} \frac{L_n(\theta)}{L_n(\hat{\theta}_n)} \leq \sup_{\|\theta-\theta_0\| \geq \delta/2} \sup_{p \in \Phi(\theta)} \prod_{i=1}^{n} n p_i \tag{1}$$

occurs with probability approaching 1, where $\Phi(\theta) = \{p : \sum_{i=1}^{n} p_i = 1, \sum_{i=1}^{n} p_i g_i(\theta) = 0, p_i \geq 0, i = 1, \dots, n\} \cup \{0\}$, and it is therefore sufficient to establish the upper-bound for the right-hand side.

By a similar argument to the proof of Lemma 1, $E_{P_0}\{g(D, \theta)\}$ is continuous in θ and we have assumed that it has a unique zero at θ_0. By the compactness of Θ, there exists some $\varepsilon > 0$ such that

$$\inf_{\|\theta-\theta_0\| \geq \delta/2} \|E_{P_0}\{g(D, \theta)\}\|_1 > \varepsilon.$$

By Assumption 1(iv), $n^{-1} \sum_{i=1}^{n} g_i(\theta)$ and $n^{-1} \sum_{i=1}^{n} \|g_i(\theta)\|_2^2$ converge uniformly in probability to $E_{P_0}\{g(D, \theta)\}$ and $E_{P_0}\{\|g(D, \theta)\|_2^2\}$ respectively. Therefore,

$$\sup_{\theta \in \Theta} \left\| \frac{1}{n} \sum_{i=1}^{n} g_i(\theta) - E_{P_0}\{g(D, \theta)\} \right\|_1 < \varepsilon/2, \quad \sup_{\theta \in \Theta} \frac{1}{n} \sum_{i=1}^{n} \|g_i(\theta)\|_2^2 < 2E_{P_0}\left\{ \sup_{\theta \in \Theta} \|g(D, \theta)\|_2^2 \right\}$$

occur with probability approaching 1. On this event,

$$\inf_{\|\theta-\theta_0\| \geq \delta/2} \left\| \frac{1}{n} \sum_{i=1}^{n} g_i(\theta) \right\|_1^2 = \inf_{\|\theta-\theta_0\| \geq \delta/2} \inf_{p \in \Phi(\theta)} \left\| \frac{1}{n} \sum_{i=1}^{n} g_i(\theta) - \sum_{i=1}^{n} p_i g_i(\theta) \right\|_1^2 > \frac{\varepsilon^2}{4}.$$

By the Cauchy-Schwarz inequality, the left hand side is bounded above by

$$\inf_{\|\theta-\theta_0\|\geq\delta/2}\ \inf_{p\in\Phi(\theta)} \left\{ \frac{1}{n}\sum_{i=1}^{n}(np_i-1)^2 \right\} \left\{ \frac{1}{n}\sum_{i=1}^{n}\|g_i(\theta)\|_2^2 \right\}.$$

Hence, there exists a strictly positive constant $\tilde{\varepsilon}$ such that

$$\inf_{\|\theta-\theta_0\|\geq\delta/2}\ \inf_{p\in\Phi(\theta)} \left\{ \frac{1}{n}\sum_{i=1}^{n}(np_i-1)^2 \right\} \geq \tilde{\varepsilon}.$$

Consider the optimization problem of maximizing $\prod_{i=1}^{n} np_i$ subject to

$$\sum_{i=1}^{n} p_i = 1, \quad \sum_{i=1}^{n}(np_i-1)^2 \geq n\tilde{\varepsilon}, \quad p_i \geq 0 \text{ for each } i = 1,\ldots,n.$$

For an element $p = (p_1,\ldots,p_n)$ in the constraint set, if p_i, p_j both exceed n^{-1} for some i, j and are unequal, replacing both with $(p_i + p_j)/2$ would strictly increase the objective while remaining in the constraint set. We deduce that for any solution to the the optimization problem, all values of p_i exceeding n^{-1} must be equal. At least one value exceeds n^{-1} due to the inequality constraint. A similar argument applies for values below n^{-1}.

For fixed $m \in \{1,\ldots,n-1\}$, we consider maximizing the objective when m values of p_i are equal to $p_+ > n^{-1}$, and the remaining $n-m$ values are equal to $p_- < n^{-1}$. We can further write $np_+ = 1 + a$, $np_- = 1 - b$, where $0 \leq a \leq n-1$, $0 \leq b \leq 1$. By taking the logarithm of the objective, we seek to maximize $m\log(1+a) + (n-m)\log(1-b)$ subject to

$$am = (n-m)b, \quad ma^2 + (n-m)b^2 \geq n\tilde{\varepsilon}.$$

For $(n\tilde{\varepsilon})/\{(n-1)^2 + \tilde{\varepsilon}\} < m < n/(1+\tilde{\varepsilon})$, the constraint set is non-empty and the solution is

$$a = \left\{ \frac{\tilde{\varepsilon}(n-m)}{m} \right\}^{1/2}, \quad b = \left[\frac{\tilde{\varepsilon}m}{n-m} \right]^{1/2}.$$

We consider sufficiently large n such that $m = 1$ lies in the permissible range. We claim that for fixed n, $m = 1$ is the value which maximizes the objective, which can now be written as

$$\left[1 + \left\{ \frac{\tilde{\varepsilon}(n-m)}{m} \right\}^{1/2} \right]^{m} \left\{ 1 - \left(\frac{\tilde{\varepsilon}m}{n-m} \right)^{1/2} \right\}^{n-m}.$$

Letting $x = \{(n-m)/m\}^{1/2}$, which is strictly decreasing in m, and taking the logarithm of the objective, it is sufficient to show that the function

$$\frac{n}{1+x^2}\log(1 + x\tilde{\varepsilon}^{1/2}) + \frac{nx^2}{1+x^2}\log\left(1 - \frac{\tilde{\varepsilon}^{1/2}}{x}\right)$$

is increasing in x. By differentiating with respect to x and simplifying, it is sufficient to show that

$$2x\left\{ \log(1 + x\tilde{\varepsilon}^{1/2}) - \log\left(1 - \frac{\tilde{\varepsilon}^{1/2}}{x}\right) \right\} - (1+x^2)\left(\frac{\tilde{\varepsilon}^{1/2}}{1 + x\tilde{\varepsilon}^{1/2}} + \frac{\tilde{\varepsilon}^{1/2}}{1 - \tilde{\varepsilon}^{1/2}/x} \right) < 0. \qquad (2)$$

The first term is equal to

$$\begin{aligned} 2x \log\left(\frac{1+x\tilde{\varepsilon}^{1/2}}{1-\tilde{\varepsilon}^{1/2}/x}\right) &= 2x\log\left\{1+\frac{\tilde{\varepsilon}^{1/2}(x^2+1)}{x(1-\tilde{\varepsilon}^{1/2}/x)}\right\} \\ &\le \frac{2\tilde{\varepsilon}^{1/2}(x^2+1)}{1-\tilde{\varepsilon}^{1/2}/x}\left(\frac{1+x\tilde{\varepsilon}^{1/2}}{1-\tilde{\varepsilon}^{1/2}/x}\right)^{-1/2} \\ &= \frac{2\tilde{\varepsilon}^{1/2}(x^2+1)}{\{(1-\tilde{\varepsilon}^{1/2}/x)(1+x\tilde{\varepsilon}^{1/2})\}^{1/2}} \end{aligned}$$

where we have used the inequality $\log(1+z) \le z(z+1)^{-1/2}$. Therefore, the left-hand side of (2) is upper-bounded by

$$\frac{\tilde{\varepsilon}^{1/2}(x^2+1)}{\{(1-\tilde{\varepsilon}^{1/2}/x)(1+x\tilde{\varepsilon}^{1/2})\}^{1/2}}\left\{2-\left(\frac{1-\tilde{\varepsilon}^{1/2}/x}{1+x\tilde{\varepsilon}^{1/2}}\right)^{1/2}-\left(\frac{1+x\tilde{\varepsilon}^{1/2}}{1-\tilde{\varepsilon}^{1/2}/x}\right)^{1/2}\right\}. \tag{3}$$

For positive z, $z+z^{-1}$ is lower-bounded by 2, with equality if and only if $z=1$. But $\tilde{\varepsilon}$ is strictly greater than 0, so

$$\left(\frac{1-\tilde{\varepsilon}^{1/2}/x}{1+x\tilde{\varepsilon}^{1/2}}\right)^{1/2}$$

cannot equal 1. Therefore, (3) is strictly less than 0, as required.

Returning to (1), we conclude that

$$\begin{aligned} \sup_{\|\theta-\theta_0\|\ge\delta/2}\ \sup_{p\in\Phi(\theta)}\prod_{i=1}^{n} np_i &\le [1+\{\tilde{\varepsilon}(n-1)\}^{1/2}]\left\{1-\left(\frac{\tilde{\varepsilon}}{n-1}\right)^{1/2}\right\}^{n-1} \\ &= [1+\{\tilde{\varepsilon}(n-1)\}^{1/2}]\exp\left[(n-1)\log\left\{1-\left(\frac{\tilde{\varepsilon}}{n-1}\right)^{1/2}\right\}\right] \\ &\le [1+\{\tilde{\varepsilon}(n-1)\}^{1/2}]\exp\{-\tilde{\varepsilon}(n-1)^{1/2}\}. \end{aligned}$$

For $0<\varepsilon^*<\tilde{\varepsilon}$, and sufficiently large n, we have a further upper-bound of $\exp\{-\varepsilon^*(n-1)^{1/2}\}$. □

Proof of Proposition 2. We work in a neighbourhood of $(0,\theta_0)$ in $\mathbb{R}^m\times\Theta$ in which Assumptions 3 and 4 hold. The function

$$E_{P_0}[\exp\{\lambda^{\mathrm{T}}g(D,\theta)\}g(D,\theta)]$$

is 0 at $(0,\theta_0)$ and the domination condition of Assumption 4 allows us to differentiate under the integral sign twice and deduce that the function is twice continuously differentiable. By the implicit function theorem, there exist a neighbourhood $\mathcal{U}\subset\Theta$ of θ_0 and a neighbourhood of $\mathcal{W}\subset\mathbb{R}^m$ of 0 such that there exists a unique twice continuously differentiable function $\lambda_0:\mathcal{U}\to\mathcal{W}$ satisfying

$$\lambda_0(\theta_0)=0,\quad E_{P_0}[\exp\{\lambda_0(\theta)^{\mathrm{T}}g(D,\theta)\}g(D,\theta)]=0$$

for all $\theta\in\mathcal{U}$. The second part of Theorem 3.1 in Csiszár (1975) implies that λ_0 is in fact the unique mapping into $\mathbb{R}^m$ which satisfies the above properties. The implicit function theorem also implies that the second derivative $\partial^2\lambda_0$ of λ_0 can be expressed as the sum and products of expectations of expressions involving λ_0, $\partial\lambda_0$, g, $\partial_\theta g$, which are all continuously differentiable in θ, and $\partial_\theta^2 g$, which satisfies the Lipschitz condition from Assumption 3, defined on a bounded set. Therefore, $\partial^2\lambda_0$ is Lipschitz continuous.□

LEMMA 1. *The function*

$$E_{P_0}\{g(D,\theta)g(D,\theta)^{\mathrm{T}}\}$$

is continuous in θ.

Proof of Lemma 1. For a fixed value $\theta^* \in \Theta$, consider a sequence $\theta_n \to \theta^*$. Define

$$f_n(d) = g(d, \theta_n)g(d, \theta_n)^{\mathrm{T}}, \quad f(d) = g(d, \theta^*)g(d, \theta^*)^{\mathrm{T}}$$

such that f_n converges pointwise to f P_0-almost everywhere and

$$\|f_n(d)\|_F \leq \sup_{\theta \in \Theta} \|g(d, \theta)g(d, \theta)^{\mathrm{T}}\|_F$$

for all n and for all values of d, where F refers to the Frobenius norm. The upper-bound is integrable, since

$$E_{P_0}\left\{\sup_{\theta \in \Theta} \|g(d, \theta)g(d, \theta)^{\mathrm{T}}\|_F\right\} = E_{P_0}\left\{\sup_{\theta \in \Theta} \|g(d, \theta)\|^2\right\} < \infty$$

by Assumption 1(iv). Therefore, we can apply the dominated convergence theorem to deduce that

$$\lim_{n \to \infty} E_{P_0}\left\{g(d, \theta_n)g(d, \theta_n)^{\mathrm{T}}\right\} = E_{P_0}\left\{g(d, \theta^*)g(d, \theta^*)^{\mathrm{T}}\right\},$$

which establishes continuity. □

LEMMA 2. *Under Assumptions 1–4, there exists a value of $\delta > 0$ such that the δ-ball around $\hat{\theta}_n$ satisfies the following properties with probability approaching 1:*

(i) contained in a neighbourhood of θ_0 satisfying the conditions of Assumptions 2 and 3 and Proposition 2.
(ii) the set of vectors $\{g_1(\theta), \ldots, g_n(\theta)\}$ span $\mathbb{R}^m$ for all values of θ.
(iii) the function $\hat{\lambda}_n(\theta)$ from Assumption 2 is the unique function mapping into $\mathbb{R}^m$ which satisfies

$$\sum_{i=1}^n \exp\{\hat{\lambda}_n(\theta)^{\mathrm{T}} g_i(\theta)\} g_i(\theta) = 0.$$

(iv) $\hat{\lambda}_n$ is twice continuously differentiable and

$$\partial\hat{\lambda}_n(\theta) = -\left\{\sum_{i=1}^n p_i(\theta)g_i(\theta)g_i(\theta)^{\mathrm{T}}\right\}^{-1}\left[\sum_{j=1}^n p_j(\theta)\{I + g_j(\theta)\hat{\lambda}_n(\theta)^{\mathrm{T}}\}\partial_\theta g_j\right]. \quad (4)$$

(v) l_n is twice differentiable with $\partial l_n(\hat{\theta}_n) = 0$ and $n^{-1}\partial^2 l_n(\hat{\theta}_n) = -\hat{\Sigma}_n^{+} = -\hat{G}_n^{\mathrm{T}}\hat{\Omega}_n^{-1}\hat{G}_n$.

Proof of Lemma 2. Consider a ball around θ_0 satisfying the conditions of Assumptions 2 and 3 and Proposition 2. By the consistency of $\hat{\theta}_n$, with probability approaching one, $\hat{\theta}_n$ is within half the radius from θ_0. Thus, we can take the ball around $\hat{\theta}_n$ of half the radius.

Assumption 1(iii) and Lemma 1 imply that there exists a neighbourhood of θ_0 where the determinant of $E_{P_0}\{g(D, \theta)g(D, \theta)^{\mathrm{T}}\}$ is bounded away from 0. By the uniform law of large numbers implied by Assumption 1(iv), $n^{-1}\sum_{i=1}^n g_i(\theta)g_i(\theta)^{\mathrm{T}}$ is positive definite for all θ in this neighbourhood with probability approaching 1. This is equivalent to the set $\{g_1(\theta), \ldots, g_n(\theta)\}$ spanning $\mathbb{R}^m$. If necessary, we shrink the ball around $\hat{\theta}_n$ to be contained in here.

The function $f_n(\lambda, \theta) = \sum_{i=1}^n \exp\{\lambda^{\mathrm{T}} g_i(\theta)\}g_i(\theta)$ is differentiable with respect to λ with partial derivative

$$\partial_\lambda f_n(\lambda, \theta) = \sum_{i=1}^n \exp\{\lambda^{\mathrm{T}} g_i(\theta)\}g_i(\theta)g_i(\theta)^{\mathrm{T}}$$

which is positive definite by the previous property. Thus, for fixed θ, $f_n(\lambda, \theta)$ is an injective mapping of λ and $\hat{\lambda}_n(\theta)$ the unique value which maps to 0.

By the uniqueness of $\hat{\lambda}_n$ and the application of the implicit function theorem to f_n at each value of $(\hat{\lambda}_n(\theta), \theta)$, $\hat{\lambda}_n$ is equal to the implicit function and is thus twice continuously differentiable. The first

derivative is

$$\partial\hat{\lambda}_n(\theta) = -\left\{\sum_{i=1}^n \exp\hat{\lambda}_n(\theta)^{\mathrm{T}}g_i(\theta)g_i(\theta)g_i(\theta)^{\mathrm{T}}\right\}^{-1}\left[\sum_{j=1}^n \exp\hat{\lambda}_n(\theta)^{\mathrm{T}}g_j(\theta)\{I_m + g_j(\theta)\hat{\lambda}_n(\theta)^{\mathrm{T}}\}\partial_\theta g_j\right]$$
$$= -\left\{\sum_{i=1}^n p_i(\theta)g_i(\theta)g_i(\theta)^{\mathrm{T}}\right\}^{-1}\left[\sum_{j=1}^n p_j(\theta)\{I_m + g_j(\theta)\hat{\lambda}_n(\theta)^{\mathrm{T}}\}\partial_\theta g_j\right].$$

We can express the log exponentially tilted empirical likelihood as

$$l_n(\theta) = \log\prod_{i=1}^n \frac{\exp\{\hat{\lambda}_n(\theta)^{\mathrm{T}}g_i(\theta)\}}{\sum_{j=1}^n \exp\{\hat{\lambda}_n(\theta)^{\mathrm{T}}g_j(\theta)\}}$$
$$= \sum_{i=1}^n\{\hat{\lambda}_n(\theta)^{\mathrm{T}}g_i(\theta)\} - n\log\sum_{j=1}^n \exp\{\hat{\lambda}_n(\theta)^{\mathrm{T}}g_j(\theta)\}$$

and we differentiate with respect to θ to obtain

$$\partial l_n(\theta) = \sum_{i=1}^n \partial(\hat{\lambda}_n^{\mathrm{T}}g_i) - n\sum_{i=1}^n \frac{\partial(\hat{\lambda}_n^{\mathrm{T}}g_i)\exp\{\hat{\lambda}_n(\theta)^{\mathrm{T}}g_i(\theta)\}}{\sum_{j=1}^n \exp\{\hat{\lambda}_n(\theta)^{\mathrm{T}}g_j(\theta)\}}$$
$$= \sum_{i=1}^n \partial(\hat{\lambda}_n^{\mathrm{T}}g_i)\{1 - np_i(\theta)\}.$$

But $p_i(\hat{\theta}_n) = 1/n$ for each $i = 1, \ldots, n$, so

$$\partial l_n(\hat{\theta}_n) = 0.$$

The second derivative of l_n is

$$\partial^2 l_n(\theta) = \sum_{i=1}^n \partial^2(\hat{\lambda}_n^{\mathrm{T}}g_i)(\theta)\{1 - np_i(\theta)\} - n\sum_{i=1}^n\{\partial(\hat{\lambda}_n^{\mathrm{T}}g_i)^{\mathrm{T}}\partial(p_i)\}(\theta).$$

Since $p_i(\hat{\theta}_n) = 1/n$ for each $i = 1, \ldots, n$, the first sum is zero at $\theta = \hat{\theta}_n$. Furthermore,

$$\partial(\hat{\lambda}_n^{\mathrm{T}}g_i)(\hat{\theta}_n) = \left(g_i^{\mathrm{T}}\partial\hat{\lambda}_n + \hat{\lambda}_n^{\mathrm{T}}\partial_\theta g_i\right)(\hat{\theta}_n)$$
$$= (g_i^{\mathrm{T}}\partial\hat{\lambda}_n)(\hat{\theta}_n)$$

since $\hat{\lambda}_n(\hat{\theta}_n) = 0$ by part (ii) and

$$\partial p_i(\hat{\theta}_n) = \left\{p_i\partial(\hat{\lambda}_n^{\mathrm{T}}g_i) - p_i\sum_{j=1}^n p_j\partial(\hat{\lambda}_n^{\mathrm{T}}g_j)\right\}(\hat{\theta}_n)$$
$$= n^{-1}(g_i^{\mathrm{T}}\partial\hat{\lambda}_n)(\hat{\theta}_n) - n^{-2}\left\{\sum_{j=1}^n g_j(\hat{\theta}_n)\right\}^{\mathrm{T}}\partial\hat{\lambda}_n(\hat{\theta}_n)$$

where the second term is zero since $\hat{\theta}_n$ is the M-estimator. We deduce from part (iii) that

$$\partial\hat{\lambda}_n(\hat{\theta}_n) = -\left\{n^{-1}\sum_{i=1}^n g_i(\hat{\theta}_n)g_i(\hat{\theta}_n)^{\mathrm{T}}\right\}^{-1}\left\{n^{-1}\sum_{j=1}^n \partial_\theta g_j(\hat{\theta}_n)\right\}$$
$$= -\hat{\Omega}_n^{-1}\hat{G}_n.$$

Putting everything together,

$$n^{-1}\partial^2 l_n(\hat\theta_n) = -\hat G_n^{\mathrm T}\hat\Omega_n^{-1}\left\{n^{-1}\sum_{i=1}^{n} g_i(\hat\theta_n)g_i(\hat\theta_n)^{\mathrm T}\right\}\hat\Omega_n^{-1}\hat G_n^{\mathrm T}$$
$$= -\hat G_n^{\mathrm T}\hat\Omega_n^{-1}\hat G_n,$$

as required. □

Proof of Theorem 2. This proof is based on the proof of Theorem 1.4.2 in Ghosh & Ramamoorthi (2003).

We make a change of variables $s = n^{1/2}(\theta - \hat\theta_n)$

$$\int_{\mathbb{R}^m}\left|p^*(s \mid D_1,\ldots,D_n) - (2\pi)^{-m/2}|\Sigma_0|^{-1/2}\exp(-0.5 s^{\mathrm T}\Sigma_0^{-1}s)\right|ds$$

where

$$p^*(s \mid D_1,\ldots,D_n) = \frac{p(\hat\theta_n + s/n^{1/2})L_n(\hat\theta_n + s/n^{1/2})}{\int p(\hat\theta_n + t/n^{1/2})L_n(\hat\theta_n + t/n^{1/2})\,dt}$$
$$= \frac{p(\hat\theta_n + s/n^{1/2})\exp\{l_n(\hat\theta_n + s/n^{1/2}) - l_n(\hat\theta_n)\}}{\int p(\hat\theta_n + t/n^{1/2})\exp\{l_n(\hat\theta_n + t/n^{1/2}) - l_n(\hat\theta_n)\}\,dt}$$

and is extended to all of $\mathbb{R}^m$ by taking the value zero outside of its original domain. Writing $C_n = \int_{\mathbb{R}^m} p(\hat\theta_n + t/n^{1/2})\exp\{l_n(\hat\theta_n + t/n^{1/2}) - l_n(\hat\theta_n)\}\,dt$, we are required to show that

$$C_n^{-1}\int_{\mathbb{R}^m}\left|p(\hat\theta_n + s/n^{1/2})\exp\{l_n(\hat\theta_n + s/n^{1/2}) - l_n(\hat\theta_n)\} - C_n(2\pi)^{-m/2}|\Sigma_0|^{-1/2}\exp\left(-s^{\mathrm T}\Sigma_0^{-1}s/2\right)\right|ds \tag{5}$$

tends in probability to 0. It is sufficient to show that

$$\mathcal{I}_1 = \int_{\mathbb{R}^m}\left|p(\hat\theta_n + s/n^{1/2})\exp\{l_n(\hat\theta_n + s/n^{1/2}) - l_n(\hat\theta_n)\} - p(\theta_0)\exp\left(-s^{\mathrm T}\Sigma_0^{-1}s/2\right)\right|ds \to 0$$

with convergence in probability, since it implies that C_n converges to $p(\theta_0)(2\pi)^{m/2}|\Sigma_0|^{1/2}$ in probability and the integral in (5) is bounded above by $\mathcal{I}_1 + \mathcal{I}_2$, where

$$\mathcal{I}_2 = \int_{\mathbb{R}^m}\left|p(\theta_0)\exp\left(-s^{\mathrm T}\Sigma_0^{-1}s/2\right) - C_n(2\pi)^{-m/2}|\Sigma_0|^{-1/2}\exp\left(-s^{\mathrm T}\Sigma_0^{-1}s/2\right)\right|ds$$
$$= \left|p(\theta_0) - C_n(2\pi)^{-m/2}|\Sigma_0|^{-1/2}\right|\int_{\mathbb{R}^m}\exp\left(-s^{\mathrm T}\Sigma_0^{-1}s/2\right)ds$$

which also converges to 0 in probability.

Let $\delta > 0$ be small enough to satisfy the conditions of Lemma 2. Let $c > 0$. We separate $\mathcal{I}_1$ into the three regions $A_1 = \{s : \|s\|_2 < c\log n^{1/2}\}$, $A_2 = \{s : c\log n^{1/2} < \|s\|_2 < \delta n^{1/2}\}$, $A_3 = \{s : \|s\|_2 > \delta n^{1/2}\}$.

We begin with A_3.

$$\int_{A_3}\left|p(\hat\theta_n + s/n^{1/2})\exp\{l_n(\hat\theta_n + s/n^{1/2}) - l_n(\hat\theta_n)\} - p(\theta_0)\exp\left(-s^{\mathrm T}\Sigma_0^{-1}s/2\right)\right|ds$$
$$\le \int_{A_3} p(\hat\theta_n + s/n^{1/2})\frac{L_n(\hat\theta_n + s/n^{1/2})}{L_n(\hat\theta_n)}\,ds + \int_{A_3} p(\theta_0)\exp\left(-s^{\mathrm T}\Sigma_0^{-1}s/2\right)ds.$$

The first integral goes to zero by Theorem 1. The second goes to zero by the tail properties of the multivariate normal distribution.

By Taylor's theorem,

$$\begin{aligned} l_n(\hat{\theta}_n + s/n^{1/2}) - l_n(\hat{\theta}_n) &= \frac{1}{2n}\partial^2 l_n(\hat{\theta}_n)(s,s) + \frac{1}{2n}\{\partial^2 l_n(\theta_s)(s,s) - \partial^2 l_n(\hat{\theta}_n)(s,s)\} \\ &= -\frac{1}{2}s^{\mathrm{T}}\hat{\Sigma}_n^{+}s + R_n(s) \end{aligned}$$

where $\theta_s = \hat{\theta}_n + (\eta s)/n^{1/2}$ for some $\eta \in [0,1]$, with the first order term vanishing due to Lemma 2. By the domination conditions of Assumption 4 and the uniqueness of λ_0 from Proposition 2, all of

$$\sup_{\theta\in\mathcal{B}_\delta(\hat{\theta}_n)} \left\|\hat{\lambda}_n(\theta) - \lambda_0(\theta)\right\|, \quad \sup_{\theta\in\mathcal{B}_\delta(\hat{\theta}_n)} \left\|\partial\hat{\lambda}_n(\theta) - \partial\lambda_0(\theta)\right\|, \quad \sup_{\theta\in\mathcal{B}_\delta(\hat{\theta}_n)} \left\|\partial^2\hat{\lambda}_n(\theta) - \partial^2\lambda_0(\theta)\right\|$$

converge to 0 in probability. For the following, let $h_i(\theta) = \lambda_0(\theta)^{\mathrm{T}} g_i(\theta)$ and $h(D,\theta) = \lambda_0(\theta)^{\mathrm{T}} g(D,\theta)$ and we suppress dependence on θ for presentational clarity

$$\frac{1}{n}\partial^2 l_n = \frac{1}{n}\sum_{i=1}^{n}\left(\partial^2 h_i\left[1 - \frac{\exp(h_i)}{E_{P_0}\{\exp h(D)\}}\right] - \frac{\exp(h_i)\partial h_i^{\mathrm{T}}}{E_{P_0}\{\exp h(D)\}}\left[\partial h_i - \frac{E_{P_0}\{\exp h(D)\partial h(D)\}}{E_{P_0}\{\exp h(D)\}}\right]\right) + o_{P_0}(1).$$

From Assumption 3 and Proposition 2, we know that for each i, $\partial^2 h_i$ satisfies a Lipschitz condition, and all other terms are continuously differentiable in θ, thus

$$\sup_{s\in A_1\cup A_2} \frac{n^{-1}\left\|\partial^2 l_n(\theta_s) - \partial^2 l_n(\hat{\theta}_n)\right\|_{op}}{\|\theta_s - \hat{\theta}_n\|_2} \leq O_{P_0}(1).$$

Now consider

$$\int_{A_1}\left|p(\hat{\theta}_n + s/n^{1/2})\exp\{l_n(\hat{\theta}_n + s/n^{1/2}) - l_n(\hat{\theta}_n)\} - p(\theta_0)\exp\left(-\frac{1}{2}s^{\mathrm{T}}\Sigma_0^{-1}s\right)\right| ds \leq J_1 + J_2$$

where

$$\begin{aligned} J_1 &= \int_{A_1} p(\hat{\theta}_n + s/n^{1/2})\left|\exp\left(-\frac{1}{2}s^{\mathrm{T}}\hat{\Sigma}_n^{+}s + R_n(s)\right) - \exp\left(-\frac{1}{2}s^{\mathrm{T}}\Sigma_0^{-1}s\right)\right| ds \\ J_2 &= \int_{A_1}\left|p(\hat{\theta}_n + s/n^{1/2}) - p(\theta_0)\right|\exp\left(-\frac{1}{2}s^{\mathrm{T}}\Sigma_0^{-1}s\right) ds. \end{aligned}$$

By consistency of $\hat{\theta}_n$ and continuity of $p(\theta)$ at θ_0, J_2 converges to 0 in probability. Furthermore,

$$\sup_{s\in A_1} R_n(s) \leq \sup_{s\in A_1}\|s\|_2^2\|\theta_s - \hat{\theta}_n\|_2\, O_{P_0}(1) \leq c^3\frac{(\log n^{1/2})^3}{n^{1/2}}O_{P_0}(1) = o_{P_0}(1)$$

and $\hat{\Sigma}_n^{+}$ converges to Σ_0^{-1} in probability by Assumption 1. Therefore, J_1 converges in probability to zero.

Next consider

$$\begin{aligned} &\int_{A_2}\left|p(\hat{\theta}_n + s/n^{1/2})\exp\{l_n(\hat{\theta}_n + s/n^{1/2}) - l_n(\hat{\theta}_n)\} - p(\theta_0)\exp\left(-\frac{1}{2}s^{\mathrm{T}}\Sigma_0^{-1}s\right)\right| ds \\ \leq &\int_{A_2} p(\hat{\theta}_n + s/n^{1/2})\exp\left\{-\frac{1}{2}s^{\mathrm{T}}\hat{\Sigma}_n^{+}s + R_n(s)\right\} ds + \int_{A_2} p(\theta_0)\exp\left(-\frac{1}{2}s^{\mathrm{T}}\Sigma_0^{-1}s\right) ds. \end{aligned}$$

The second integral is bounded above by $p(\theta_0)\exp\{-\zeta(c\log n^{1/2})^2/2\}\mathrm{vol}(A_2)$ where $\zeta > 0$ is the smallest eigenvalue of Σ_0^{-1}. For $n^{1/2} > e$, $(\log n^{1/2})^2 > \log n^{1/2}$, so we can further upper-bound the second integral by

$$Kp(\theta_0)\frac{n^{m/2}}{n^{\zeta c^2/4}}$$

where $K > 0$ is a constant. For sufficiently large c, this tends to 0 as n tends to infinity.

For the first integral, since $\|\theta_s - \hat\theta_n\|_2 < \delta$ for all $s \in A_2$, we have

$$\sup_{s\in A_2} \frac{|R_n(s)|}{\|s\|_2^2} \le \delta\, O_{P_0}(1).$$

Therefore, for any $\varepsilon > 0$, we can choose sufficiently small δ to ensure that

$$\mathrm{pr}\left\{ |R_n(s)| < \frac{1}{4} s^{\mathrm{T}} \hat\Sigma_n^{+} s \text{ for all } s \in A_2 \right\} > 1 - \varepsilon$$

for all sufficiently large n. Hence, with probability greater than $1-\varepsilon$,

$$\int_{A_2} p(\hat\theta_n + s/n^{1/2}) \exp\left\{ -\frac{1}{2} s^{\mathrm{T}}\hat\Sigma_n^{+} s + R_n(s)\right\} ds$$
$$\le \sup_{s\in A_2} p(\hat\theta_n + s/n^{1/2}) \int_{A_2} \exp\left(-\frac{1}{4} s^{\mathrm{T}} \hat\Sigma_n^{+} s\right) ds$$

which converges to zero in probability. □

Proof of Theorem 3. Using the same notation as the proof of Theorem 2, we claim that

$$\int_{\mathbb{R}^m} \|s\,\{p^*(s \mid D_1,\ldots,D_n) - (2\pi)^{-m/2}|\Sigma_0|^{-1/2}\exp(-0.5 s^{\mathrm{T}}\Sigma_0^{-1}s)\}\|_2\, ds \to 0$$

with convergence in probability. This is similar to what was proved in Theorem 2, but there is now an additional factor of $\|s\|_2$ in the integrand. The claim implies that

$$\left\| \int_{\mathbb{R}^m} s\,\{p^*(s \mid D_1,\ldots,D_n) - (2\pi)^{-m/2}|\Sigma_0|^{-1/2}\exp(-0.5 s^{\mathrm{T}}\Sigma_0^{-1}s)\}\, ds \right\|_2 \to 0$$

with convergence in probability, but the second term within the norm is equal to the mean of a mean zero multivariate normal distribution. Thus,

$$n^{1/2}(\theta_n^* - \hat\theta_n) = \int_{\mathbb{R}^m} s\, p^*(s \mid d_1,\ldots,d_n)\, ds \to 0$$

with convergence in probability. The second assertion follows from this along with the asymptotic normality of $\hat\theta_n$ stated in §2.1.

It remains to prove the initial claim. Since $\int_{\mathbb{R}^m} \|s\|_2 \exp(-0.5 s^{\mathrm{T}}\Sigma_0^{-1}s)\, ds < \infty$, we can argue similarly to the proof of Theorem 2 that it is sufficient to show

$$\int_{\mathbb{R}^m} \|s\{p(\hat\theta_n + s/n^{1/2}) \exp\{l_n(\hat\theta_n + s/n^{1/2}) - l_n(\hat\theta_n)\} - p(\theta_0)\exp(-s^{\mathrm{T}}\Sigma_0^{-1}s/2)\}\|_2\, ds \to 0$$

with convergence in probability. As before, we decompose the integral into the three regions A_1, A_2 and A_3. For A_3,

$$\int_{A_3} \|s\{p(\hat\theta_n + s/n^{1/2}) \exp\{l_n(\hat\theta_n + s/n^{1/2}) - l_n(\hat\theta_n)\} - p(\theta_0)\exp(-s^{\mathrm{T}}\Sigma_0^{-1}s/2)\}\|_2\, ds$$
$$\le \int_{A_3} \|s\|_2\, p(\hat\theta_n + s/n^{1/2}) \frac{L_n(\hat\theta_n + s/n^{1/2})}{L_n(\hat\theta_n)}\, ds + \int_{A_3} \|s\|_2\, p(\theta_0)\exp(-s^{\mathrm{T}}\Sigma_0^{-1}s/2)\, ds.$$

Changing variables back to θ, the first integral on the right hand side is equal to

$$\int_{\|\theta-\hat\theta_n\|_2>\delta} n^{(m+1)/2}\|\theta - \hat\theta_n\|_2\, p(\theta) \frac{L_n(\theta)}{L_n(\hat\theta_n)}\, d\theta.$$

But

$$\int_{\|\theta-\hat\theta_n\|_2>\delta} \|\theta-\hat\theta_n\|_2\, p(\theta)\, d\theta \le \int_{\|\theta-\hat\theta_n\|_2>\delta} (\|\theta\|_2 + \|\hat\theta_n\|_2)\, p(\theta)\, d\theta,$$

and the right hand side is stochastically bounded by the finite moment assumption. Thus, by applying Theorem 1, the first integral tends to zero in probability. The second integral also tends to zero in probability by the tail properties of the multivariate normal distribution.

Furthermore,

$$\int_{A_1} \|s\|_2 \exp(-s^{\mathrm{T}}\Sigma_0^{-1}s/2)\, ds = O_{P_0}(1) \quad \text{and} \quad \int_{A_2} \|s\|_2 \exp\left(-s^{\mathrm{T}}\Sigma_0^{-1}s/4\right) ds \to 0$$

with convergence in probability, from which we can deduce that the integrals for A_1 and A_2 will also converge to 0 in probability using the same arguments as the proof of Theorem 2. □

Proof of Theorem 4. Theorem 2 implies L^1 convergence of the full posterior as $n \to \infty$

$$\int_{\Theta} \left| p(\theta \mid D_1, \ldots, D_n) - p_{\hat{\theta}_n, n^{-1}\Sigma_0}(\theta) \right| d\theta \to 0$$

with convergence in probability, where $\Theta \subset \mathbb{R}^m$ is the parameter space of θ, $p_{\hat{\theta}, n^{-1}\Sigma_0}$ is the density of $\mathcal{N}(\hat{\theta}_n, n^{-1}\Sigma_0)$, $\hat{\theta}_n = (\hat{\alpha}_n, \hat{\beta}_n, \hat{\rho}_n, \hat{\gamma}_n)$ and $\Sigma_0 = \lim_{n\to\infty} \mathrm{var}_{P_0}(n^{1/2}\hat{\theta}_n)$. It remains to show the corresponding result for the marginal posterior. Let $m_1 = \dim(\alpha) + \dim(\beta) + \dim(\rho)$, so that $(\alpha, \beta, \rho) \in \mathbb{R}^{m_1}$, and let $m_2 = \dim(\gamma)$, so $m_1 + m_2 = m$. The posterior density $p(\theta \mid d_1, \ldots, d_n)$ is assigned the value 0 outside of Θ.

$$\begin{aligned}
\int_{\Gamma} \left| p(\gamma \mid D_1, \ldots, D_n) - p_{\hat{\gamma}_n, n^{-1}V_0}(\gamma) \right| d\gamma &= \int_{\Gamma} \left| \int_{\mathbb{R}^{m_1}} p(\theta \mid D_1, \ldots, D_n) - p_{\hat{\theta}_n, n^{-1}\Sigma_0}(\theta)\, d\alpha\, d\beta\, d\rho \right| d\gamma \\
&\leq \int_{\Gamma} \int_{\mathbb{R}^{m_1}} \left| p(\theta \mid D_1, \ldots, D_n) - p_{\hat{\theta}_n, n^{-1}\Sigma_0}(\theta) \right| d\alpha\, d\beta\, d\rho\, d\gamma \\
&\leq \int_{\mathbb{R}^{m_2}} \int_{\mathbb{R}^{m_1}} \left| p(\theta \mid D_1, \ldots, D_n) - p_{\hat{\theta}_n, n^{-1}\Sigma_0}(\theta) \right| d\alpha\, d\beta\, d\rho\, d\gamma \\
&= \int_{\Theta} \left| p(\theta \mid D_1, \ldots, D_n) - p_{\hat{\theta}_n, n^{-1}\Sigma_0}(\theta) \right| d\theta \\
&+ \int_{\mathbb{R}^m \setminus \Theta} p_{\hat{\theta}_n, n^{-1}\Sigma_0}(\theta)\, d\theta.
\end{aligned}$$

The first term tends in probability to 0 by Theorem 2. This implies that

$$\int_{\Theta} p_{\hat{\theta}_n, n^{-1}\Sigma_0}(\theta)\, d\theta \to 1$$

with convergence in probability, so the second term also tends in probability to 0. □

REFERENCES

CSISZÁR, I. (1975). I-divergence geometry of probability distributions and minimization problems. *Annals of Probability* **3**, 146–158.

GHOSH, J. & RAMAMOORTHI, R. (2003). *Bayesian Nonparametrics*. New York: Springer-Verlag.

[*Received. Editorial decision on*]
